# Carbamazepine-Mediated Adverse Drug Reactions: CBZ-10,11-epoxide but Not Carbamazepine Induces the Alteration of Peptides Presented by HLA-B∗15:02

**DOI:** 10.1155/2018/5086503

**Published:** 2018-09-13

**Authors:** Gwendolin S. Simper, Gia-Gia T. Hò, Alexander A. Celik, Trevor Huyton, Joachim Kuhn, Heike Kunze-Schumacher, Rainer Blasczyk, Christina Bade-Döding

**Affiliations:** ^1^Institute for Transfusion Medicine, Hannover Medical School, Carl-Neuberg-Str. 1, 30625 Hannover, Germany; ^2^Department of Cellular Logistics, Max Planck Institute for Biophysical Chemistry, Am Fassberg 11, 37077 Göttingen, Germany; ^3^Institute for Laboratory and Transfusion Medicine, Heart and Diabetes Center North Rhine-Westphalia, Ruhr University Bochum, Georgstraße 11, 32545 Bad Oeynhausen, Germany

## Abstract

Among patients treated with the anticonvulsive and psychotropic drug carbamazepine (CBZ), approximately 10% develop severe and life-threatening adverse drug reactions. These immunological conditions are resolved upon withdrawal of the medicament, suggesting that the drug does not manifest in the body in long term. The HLA allele B∗15:02 has been described to be a genomic biomarker for CBZ-mediated immune reactions. It is not well understood if the immune reactions are triggered by the original drug or by its metabolite carbamazepine-10,11-epoxide (EPX) and how the interaction between the drug and the distinct HLA molecule occurs. Genetically engineered human B-lymphoblastoid cells expressing soluble HLA-B∗15:02 molecules were treated with the drug or its metabolite. Functional pHLA complexes were purified; peptides were eluted and sequenced. Applying mass spectrometric analysis, CBZ and EPX were monitored by analyzing the heavy chain and peptide fractions separately for the presence of the drug. This method enabled the detection of the drug in a biological situation post-pHLA assembly. Both drugs were bound to the HLA-B∗15:02 heavy chain; however, solely EPX altered the peptide-binding motif of B∗15:02-restricted peptides. This observation could be explained through structural insight; EPX binds to the peptide-binding region and alters the biochemical features of the F pocket and thus the peptide motif. Understanding the nature of immunogenic interactions between CBZ and EPX with the HLA immune complex will guide towards effective and safe medications.

## 1. Introduction

The highly polymorphic human leukocyte antigen (HLA) system plays a central role within self-/non-self-recognition in adaptive immune responses through the presentation of peptides originating from self or pathogenic sources. CD8^+^ T cells scan peptide-HLA class I (pHLA) complexes on the surface of virtually every cell type. The peptide-binding region (PBR) of an HLA molecule can be subdivided into compartments of distinct amino acids (AAs) designated as pockets, where each pocket binds distinct AA side chains of a given peptide [1]. This dynamic binding between HLA pockets and peptides results in HLA allele-specific peptide-binding motifs, determining the pool of bound peptides [2–4]. The peptide specificity of HLA molecules enables exposure of a high pHLA complex variety that determines allele-specific footprints.

Certain HLA class I alleles are associated with hypersensitive reactions to drugs [5]. Occurring in more than 7% of the general population, adverse drug reactions (ADRs) represent a major problem in public health associated with global morbidity and mortality [6]. ADRs are defined as unintended and harmful reactions to drugs. They arise despite appropriate application, such as standard dosage, adequate route of administration, and usual range of application (WHO, 1972). ADRs can become life-threatening and expensive complications [7–9] that are often underestimated due to underdiagnosis and underreporting [10, 11]. Predictable and dose-dependent type A ADRs can be distinguished from dose-independent idiosyncratic type B ADRs [12]. The emergence of type B ADRs is mostly not well understood, yet, but is often linked to immune reactions [13–15]. Most frequent symptoms are skin rashes; however, in some cases, liver and blood cells can be affected [13, 16]. Cutaneous hypersensitivity reactions comprise mild maculopapular eruption (MPE) and drug reaction with eosinophilia and systemic symptoms (DRESS), as well as the more harmful Stevens-Johnson syndrome (SJS) and toxic epidermal necrolysis (TEN) being lethal in about 48% of cases [17, 18]. Thus, the enormous relevance to assess the mechanisms of currently unpredictable ADRs becomes obvious.

Several studies identified allelic HLA variants to be associated with distinct cutaneous ADRs [13, 19]. A multiplicity of HLA-associated ADRs is CD8^+^ T cell-mediated reactions, for example, abacavir- or carbamazepine- (CBZ-) induced ADRs [20–23], implying an alteration of the accessible pHLA surface through immune interference by the drug.

Three models propose a mechanism of T cell activation via drugs. The hapten/prohapten model assumes that small chemicals provoke immune responses after reacting with nucleophilic groups on proteins [24–28], eluding the dogma of small molecules (<1 kDa) not being antigenic [29]. This model states that covalent binding of a reactive drug (hapten) or a reactive metabolite of a chemically inert drug (prohapten) to a high molecular weight protein turns it immunogenic following presentation by HLA molecules [28, 29]. A well-studied example is the immunological reaction induced by penicillin administration, where binding of the drug to lysine side chains of HLA-bound peptides triggers a B and T cell response [30–33]. In contrast, a metabolite of the prohapten sulfamethoxazole bound to presented peptides triggers T cell proliferation [34].

Due to the finding that following sulfamethoxazole treatment fixed cells still stimulate T cells, even though fixation hinders antigen presentation, the second model was proposed. Sulfamethoxazole could be washed off, indicating a transient interaction but not a permanent binding [35, 36]. According to this pharmacological interaction (p-i) model, drugs can bind noncovalently but specifically to immune receptors causing an immediate T cell response [37, 38]. This model explains a direct and fast response of the adaptive immune system independent from processing or metabolism [39].

Finally, the altered repertoire model suggests that the immune reaction is triggered by a shift in the self-peptide repertoire. This model was proven for the nucleoside reverse transcriptase inhibitor abacavir triggering hypersensitivity reactions in HLA-B∗57:01-positive patients [5, 40]. First described in 2002 by Mallal et al. [41] and Hetherington et al. [42], the association of abacavir-induced ADRs with HLA-B∗57:01 was later understood as abacavir being bound to the F pocket within the PBR during pHLA maturation. This alteration of the PBR features leads to a different peptide repertoire being presented to the immune system, mimicking a pathogenic condition to the surveying immune cells [5, 43].

The tricyclic anticonvulsant CBZ, developed by Schindler in 1953, was first applied as a treatment for depressions and psychosis [44–46]. Later, its positive effect on trigeminal neuralgia and epilepsy was described [44, 47]. Since its first approval for therapy 50 years ago, CBZ became the standard drug against partial onset seizures [44, 48]. Shortly after the approval, first side effects were observed including cutaneous ADRs [45, 49]. In 2004, Chung et al. [50] revealed the association of HLA-B∗15:02 with CBZ-induced SJS in Han Chinese. In Caucasians and some Asian populations, HLA-A∗31:01 was found to be associated with cutaneous ADRs with milder symptoms [51–53]. Further studies confirmed that carriers of HLA-B∗15:02 develop severe SJS/TEN upon treatment with CBZ [17, 54–56] emerging after 15 days as determined in the RegiSCAR study [57].

The half-life of CBZ is approximately 35 h for a single oral dose and decreases to 10–20 h in long-term therapy [58]. CBZ is metabolized by cytochrome P450 enzymes resulting in potentially reactive substances, for example, CBZ-10, 11-epoxide (EPX), that are detoxified afterwards [17]. The drug triggers its own metabolism via autoinduction [58]. However, CBZ-induced ADRs are described to be independent of polymorphisms in P450 in Han Chinese [50] and epoxide hydrolase in Caucasians [59].

In the literature, modifications of the presented peptides and alterations of the peptidome were discussed [5, 60]. It has been shown that not only CBZ but also derivatives, most prominently EPX, can bind soluble immobilized HLA-B∗15:02 [61]. As described in the International Consensus on drug allergy [62], ADRs such as MPE, DRESS, SJS, and TEN can emerge within no less than one hour but commonly arise after several days of treatment. Thus far, it is unclear whether CBZ, EPX, or both substances are contributing to HLA-associated hypersensitivity reactions. Interestingly, He et al. [63] showed an increasing risk of SJS/TEN that is dependent on the epoxide hydrolase 1 (EPHX1) polymorphism, yet no influence of CYP3A4, multidrug resistance protein 1, FAS, and SCN1a polymorphism in Chinese Han population could be observed.

The aim was to understand the obstacle of CBZ hypersensitivity on a molecular basis. To this end the biophysical interaction between CBZ, EPX, and pHLA-B∗15:02 complexes and its bound peptides was analyzed by mass spectrometry and computational modelling of the pHLA-B∗15:02/EPX structures. This method allowed for the first time deep insight in the mechanism that triggers the T cell responses following CBZ application. Understanding the pathogenesis of life-threatening CBZ hypersensitivity in HLA-B∗15:02 carriers would support medication safety and cost-effective personalized treatment [64].

## 2. Material and Methods

### 2.1. Cell Lines

All cell lines were maintained at 37°C and 5% CO_2_. The human B-lymphoblastoid cell line LCL721.221 (HLA class I^−^) was cultured in RPMI 1640 (Lonza, Basel, Switzerland) supplemented with 10% heat-inactivated fetal calf serum (FCS, Lonza, Basel, Switzerland), 2 mM L-glutamine (c.c.pro, Oberdorla, Germany), 100 U/ml penicillin, and 100 *μ*g/ml streptomycin (c.c.pro, Oberdorla, Germany). The human embryonal kidney cell line HEK293T was maintained in DMEM (Lonza, Basel, Switzerland) supplemented with 10% heat-inactivated FCS, 2 mM L-glutamine, 100 U/ml penicillin, 100 *μ*g/ml streptomycin, and 1 mg/ml Geneticin® (Life Technologies, Carlsbad, USA).

### 2.2. Cloning of B∗15:02 into the Lentiviral pRRL.PPT.SFFV.mcs.pre Vector

The sequence for soluble HLA-B∗15:02 was generated via site-directed mutagenesis in five steps from the vector pcDNAv3.1/B∗35:01 (exons 1–4) according to Celik et al. [65]. The success of each mutagenesis was verified by sequencing. The insert was subcloned into the lentiviral vector pRRL.PPT.SFFV.mcs.pre and sequenced [66].

### 2.3. Transduction of LCL721.221 Cells for sHLA Production

Utilizing Lipofectamine® 2000 (Life Technologies, Carlsbad, USA), HEK293T cells were transfected with the target plasmid (10 *μ*g/5 × 10^6^ cells) along with the packaging and envelope vectors psPAX2 and pmD2.G (5 *μ*g/5 × 10^6^ cells) as described by Bade-Doeding et al. [66]. LCL721.221 cells were transduced with the viral particles. Expression of trimeric sHLA molecules was confirmed by HLA class I-specific ELISA [66].

### 2.4. Production of Soluble pHLA and pHLA/CBZ Complexes

Recombinant LCL721.221/sB∗15:02 cells were cultured at a cell density of 1 × 10^6^ cells/ml in the absence or presence of 25 *μ*g/ml CBZ (Toronto Research Chemicals, Toronto, Canada) or EPX (Toronto Research Chemicals, Toronto, Canada), and supernatant was collected biweekly. Following centrifugation (300 ×g, 10 min), cells and cellular debris were discarded and supernatant was filtered through a 0.45 *μ*m membrane (Millipore, Schwalbach, Germany) and adjusted to pH 8.0. sHLA-B∗15:02 (w/o drug, w/ CBZ, and w/ EPX) complexes were purified via affinity chromatography using an NHS-activated HiTrap column (Life Technologies, Carlsbad, USA) coupled to an anti-HLA class I antibody (clone W6/32). Elution of peptides was performed with 100 mM glycine/HCl buffer pH 2.7.

### 2.5. Mass Spectrometric Sequencing of the Presented Peptides

Following elution, low-affinity peptides were separated from the HLA molecules by centrifugation through a membrane with a cutoff of 10 kDa (Millipore, Schwalbach, Germany). Afterwards, treatment of the remaining trimeric HLA molecules in the retentate with 0.1% trifluoroacetic acid was used to elute peptides of high affinity. Subsequently, the peptides were sequenced using a nano-LC-ESI-MS/MS (LTQ Orbitrap) (Thermo Fisher, Waltham, USA).

### 2.6. Measuring of CBZ and EPX in the Trimeric pHLA Complex and after Separation of Low- and High-Affinity Peptides

Functional trimeric affinity-purified HLA complexes were analyzed for the presence of CBZ or EPX via mass spectrometry. For this purpose, CBZ and EPX were separated on a 2.1 × 50 mm reverse phase column (Waters, ACQUITY UPLC BEH Phenyl, 1.7 *μ*m) maintained at 50°C using an ultraperformance liquid chromatography system directly coupled to a Waters TQ electrospray ionization-tandem mass spectrometry (TQD). An injection volume of 1.0 *μ*l sample was used for each analysis. The flow rate of the mobile phase was set at 0.5 ml/min. The gradient program was as follows: isocratic flow of 75/25 water/methanol containing 0.1% formic acid and 2 mmol/l ammonium acetate was performed for 1.0 min, followed by a linear gradient over 1.3 min of 5%/95% water/methanol containing also 0.1% formic acid and 2 mmol/l ammonium acetate. After the isocratic elution of 95% methanol for 0.5 min, the mobile phase was revered to the initial state and the run was ended at 3.5 min. The TQD was operated in an electrospray-positive ionization mode. The system controls of the devices and data acquisition were performed using MassLynx NT 4.1 software. Data processing was performed by the MassLynx QuanLynx program. Nitrogen was used as the nebulizing gas, and argon was used as the collision gas. Instrument setting was as follows: capillary voltage, 0.42 kV; source temperature, 110°C; desolvation temperature, 480°C; and collision gas pressure, 2.3 × 10^−3^. Sample analysis was performed in the multiple reaction monitoring (MRM) mode of the instrument (Table 1). Sample cone voltage, collision energy, dwell time, and mass transitions for all compounds are listed in Table 1. The mass transition which was used for quantification of CBZ and EPX (first transition) is written in bold [67]. Accordingly, low and high binding peptides as well as the appropriate retentate were analyzed for the presence of the drugs.

### 2.7. Modelling

The 9-mer peptide SQATPHSSY could solely be detected to bind to HLA-B∗15:02 in the absence of drugs and was chosen for modelling of the pHLA-B∗15:02 complex. Exclusively eluted from the functional B∗15:02 complex following treatment with EPX, the 11-mer VSQqKLQAEAQ was selected for modelling the pHLA-B∗15:02/EPX complex. SWISS-MODEL [68] was first used to generate a homology model of B∗15:02 from the structure of B∗15:01 (1XR8). We then used RosettaLigand [69] to dock EPX to the heavy-chain peptide-binding groove creating 200 models and selecting the best model with the lowest interface_delta score.

Rosetta FlexPepDock [70] was used for peptide docking on models with and without EPX. A total of 200 models were created and then ranked based on their Rosetta generic full-atom energy score.

## 3. Results

AA length of peptides presented by HLA-B∗15:02 is similar to other HLA class I molecules.

Since severe CBZ-mediated ADRs are associated with the HLA allele B∗15:02, but the molecular mechanism still remains unclear, we aimed to compare the impact of CBZ and its main metabolite EPX on peptide presentation of B∗15:02. In order to analyze the peptidome of B∗15:02, the human lymphoblastoid cell line LCL721.221 was transduced with lentiviral vectors encoding for sB∗15:02 and functional HLA complexes were purified via affinity purification. Low and high binding peptides were separated for subsequent mass spectrometric analysis.

**Figure 1:**
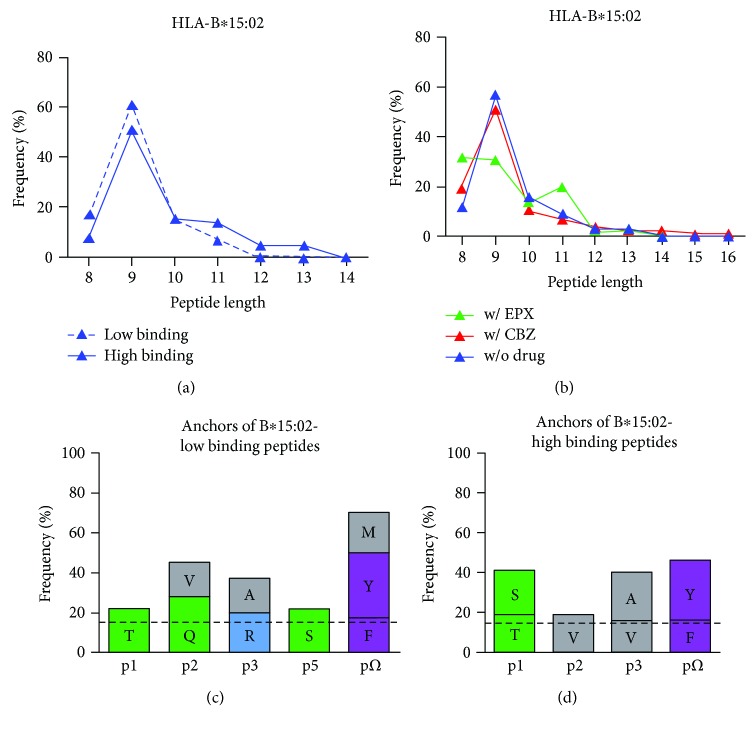
Characteristics of peptides presented by HLA-B∗15:02. (a) Length distribution of low and high binding peptides presented by HLA-B∗15:02. (b) Length distribution of peptides presented by HLA-B∗15:02 without treatment (blue), after incubation with CBZ (red) and EPX (green). (c) Preferred AAs of low binding peptides presented by HLA-B∗15:02. (d) Preferred AAs of high binding peptides presented by HLA-B∗15:02. All AAs with a frequency higher than 15% (dashed line) are given for position p1-p3, p5, and pΩ. AAs are distinguished into polar positive (blue), polar neutral (green), nonpolar aliphatic (grey), and nonpolar aromatic (purple) AAs.

The peptides presented by B∗15:02 exhibited a length distribution from 8 to 13 AAs (Figure 1(a)). 85% of all peptides featured a length of 8–10 AAs. As expected for HLA class I molecules, 9-mers were found most frequent.

### 3.1. HLA-B∗15:02 Has a Preference for Nonpolar Aromatic AAs Tyrosine and Phenylalanine at pΩ

The ability of HLA molecules to present specific peptides is substantially governed by the AAs forming the PBR. Nevertheless, a variety of different peptides can bind to the PBR of a specific HLA allele, mainly characterized by shared anchor AAs. High and low binding peptides of B∗15:02 were anchored by AAs deviating from each other. Three AAs dominated the pΩ residue for B∗15:02-low binding peptides: nonpolar aromatic tyrosine (33%) and phenylalanine (17%), as well as nonpolar aliphatic methionine (20%) (Figure 1(c)). Similarly, high binding peptides were anchored mainly by nonpolar aromatic AAs tyrosine (30%) and phenylalanine (16%) at pΩ but not methionine (11%) (Figure 1(d)). At p1 of low binding peptides, threonine (22%) was most frequent whereas in high binding peptides, this position was occupied by both nonpolar and neutral AAs serine (22%) and threonine (19%). At p2, preferred AAs constitute glutamine (28%) or valine (17%) in low binding and valine (19%) in high binding peptides. Alanine (17%) and arginine (20%) were found most frequent at p3 of B∗15:02-low binding peptides, while nonpolar aliphatic AAs alanine (24%) and valine (16%) were most frequent at this position in high binding peptides. P5 of low binding peptides was dominated by serine (22%).

### 3.2. CBZ as well as EPX Bind to B∗15:02 Heavy Chain

**Figure 2:**
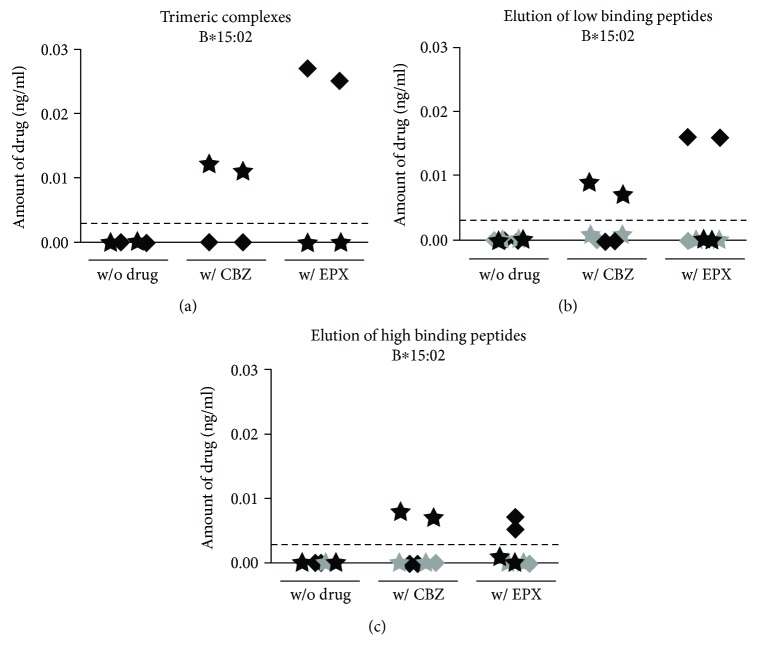
CBZ and EPX bind to the HLA heavy chain and not to the peptides. sB∗15:02 molecules were produced in the HLA class I-negative LCL721.221 cell line without treatment and in the presence of CBZ or EPX and purified via affinity chromatography. The amount of the drugs was measured mass spectrometrically (CBZ shown as ★, EPX shown as ◆). The threshold for drug detections is represented by a dashed line. The trimeric complexes (a) were analyzed as well as retentate and peptide fractions after separation of low (b) and high binding peptides (c). The amount of drug measured in the HLA heavy-chain fraction is displayed in black; the amount measured in the peptide fraction is shown in grey.

CBZ-mediated ADRs might be caused by covalent binding of the drug itself or a reactive metabolite to a higher molecular weight protein that is subsequently presented by B∗15:02. Alternatively, the drug can interact with both HLA molecule and T cell receptor or bind to the PBR and alter the peptide repertoire. After incubation of LCL721.221 cells producing sB∗15:02 with CBZ or EPX, functional molecules were purified using affinity chromatography. The resulting fraction containing the trimeric molecules was analyzed for the presence of CBZ and EPX via mass spectrometry. Not only CBZ but also EPX was able to bind to B∗15:02 (Figure 2(a)). Both molecules were detected in the fraction with purified trimeric molecules of B∗15:02. To exclude the p-i model, the altered repertoire model, or the hapten/prohapten model, low and high binding peptides were separated and analyzed for the presence of CBZ and EPX. The successful separation of heavy chain, light chain, and peptides was ascertained by Western blot and nano-LC-ESI-MS/MS analysis. After separation of low binding peptides, the drugs remained in the retentate containing HLA heavy and light chains (Figure 2(b)). Similarly, CBZ and EPX were absent in high binding peptide fractions but present in the retentate (Figure 2(c)).

### 3.3. Treatment with EPX Alters the Peptide-Binding Motif of HLA-B∗15:02

**Figure 3:**
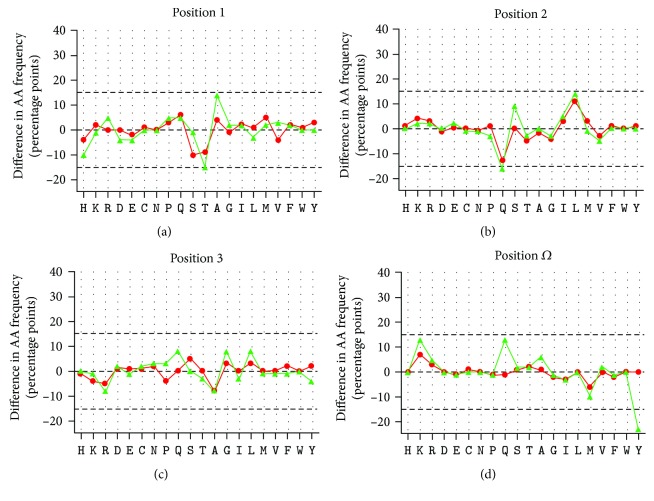
Alteration of peptides bound to B∗15:02/CBZ or B∗15:02/EPX complexes. The alterations of AA frequency after treatment with CBZ (red) and EPX (green) are depicted at p1 (a), p2 (b), p3 (c), and pΩ (d). The graphs for CBZ and EPX were adjusted to the data of AA frequencies obtained without drug treatment (black). The data have been obtained from triplicate experiments.

Since both molecules, CBZ and EPX, were able to bind to the heavy chain of B∗15:02, the effect of either substance on the peptide-binding motif was evaluated. Treatment with EPX affected peptide presentation for B∗15:02 (Figure 3). Threonine at p1 was exchanged for alanine after treatment with EPX. The frequency of threonine decreased from 19% to 5%, whereas the frequency of alanine increased from 4% to 18%. Treatment with CBZ did not induce such alterations, although the frequency of serine and threonine was slightly reduced after treatment with CBZ. At p2 of the bound peptides, treatment with CBZ or EPX had a comparable effect on the rate of AAs. Glutamine was decreased from 24% to 11% or 8%, respectively, in favor of leucine increasing from 9% to 20% or 23%. Alterations at p3 after treatment with the drugs were faint. At pΩ of B∗15:02 restricted peptides in the presence of EPX, the frequency of polar AAs glutamine and lysine increased (from 4% to 17% for glutamine and to 18% for lysine), chargeable to the frequency of tyrosine (decreasing from 32% to 9%) and methionine (decreasing from 16% to 6%). Treatment with CBZ had minimal effects on AA frequencies at pΩ.

### 3.4. Treatment with EPX Leads to a Decrease in 9-mers Presented by HLA-B∗15:02

Treatment with CBZ did not affect the length of the peptides presented by B∗15:02. The peptides eluted from B∗15:02 after treatment with EPX showed an altered pattern (Figure 1(b)). The amount of 9-mers was decreased (from w/o drug 57%, w/ CBZ 51% to w/ EPX 31%), instead longer, as well as shorter peptides were more frequent. The abundance of 8-mers (32%) and 9-mers (31%) was similar. The amount of 10-mers remained consistent after treatment with EPX (w/o drug 16%, w/ CBZ 11%, and w/ EPX 14%), but the amount of 11-mers increased (w/o drug 9%, w/ CBZ 7%, and w/ EPX 20%).

### 3.5. Deamidation Is Increased at p4 after Treatment with EPX for HLA-B∗15:02

**Figure 4:**
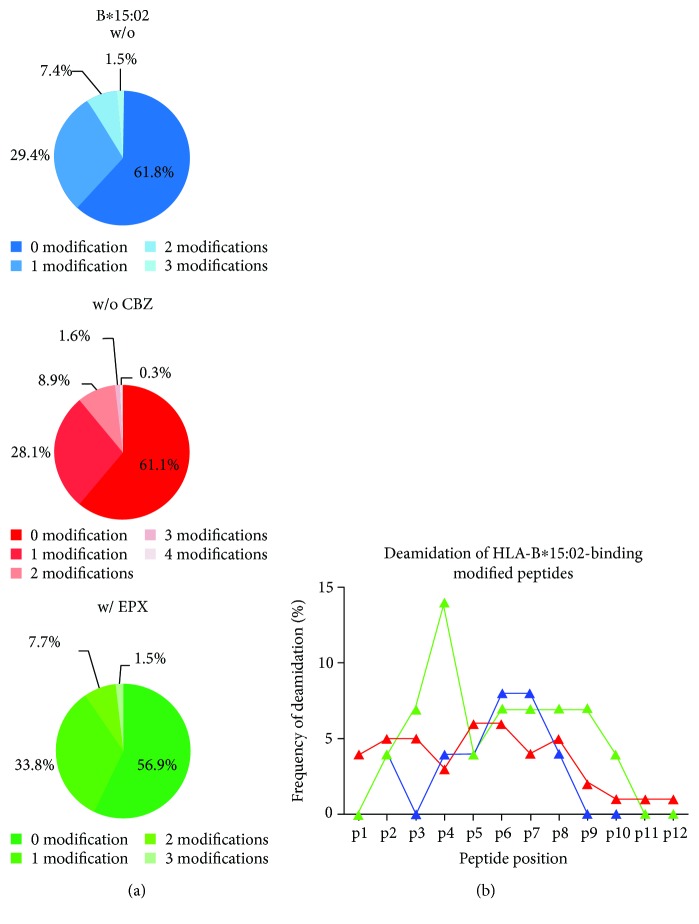
Deamidation at p4 is altered after treatment with EPX for B∗15:02. (a) Given is the amount of modified peptides presented by B∗15:02 w/o treatment (blue), after treatment with CBZ (red) and EPX (green). (b) Frequency of deamidation of modified peptides derived from B∗15:02.

Since HLA class I molecules present cytosolic peptides, they are posttranslationally modified to some extent. Analysis of the peptides presented by B∗15:02 revealed that 40% display one or more modifications, with a single modification being the most frequent (28–34%) (Figure 4(a)). The highest number of modified peptides was observed after treatment with EPX. Interestingly, only the number of peptides with one modification was increased. Deamidation, n-terminal acetylation, and carbamidomethylation were some of the modifications found in these peptides. Comparing the position of deamidation revealed that after treatment with EPX the AA at p4 was deamidated more frequently than without drug incubation and after CBZ treatment (Figure 4(b)). The rate of deamidation at this position increased from 4% (w/o drug) or 3% (w/ CBZ) to 14% (w/ EPX).

### 3.6. EPX Binds to the F Pocket of HLA-B∗15:02

**Figure 5:**
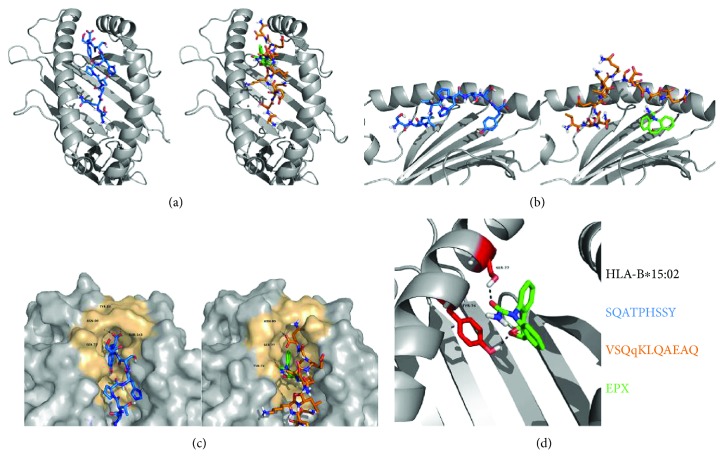
Modelling of peptides bound to empty and EPX bound HLA∗B15:02. The homology model of HLA-B∗15:02 (grey) is based on the structure of HLA-B∗15:01 (1XR8). The peptide SQATPHSSY (blue) could be solely detected without treatment, whereas following treatment with EPX (green), the peptide VSQqKLQAEAQ (orange) was detected. (a) Top view on the PBR of the pHLA complex in the absence and presence of EPX. (b) Side view on the PBR, the alpha helix of the alpha 2 domain is not shown. (c) Surface model of peptides and EPX binding to the F pocket (colored brownish). The AAs interacting with the peptide or the drug via hydrogen bridge bonds are depicted. (d) The oxygen atom in the carboxamide moiety of EPX interacts with two AAs of the F pocket.

To understand how EPX might occupy the PBR for alteration of the C-terminal peptide anchor, we modelled EPX in the PBR of B∗15:02 unbound to a peptide. Unliganded structures are generally more difficult to dock into because of their unknown binding sites. The large peptide-binding groove of HLA molecules and robust docking performance of RosettaLigand could however be used to generate a model of EPX binding that could be used in further peptide docking experiments. Similar poses were found in the top 10 models with the best model having an interface delta score of −10.75, a score that is comparable to the native binding pose of the RosettaLigand test dataset MR3 in T4 lysozyme. The EPX-binding pocket was determined by analysis through the PISA web server (http://www.ebi.ac.uk/pdbe/pisa/) and was exclusively comprised of residues previously described to be part of the F pocket [1]: Y74, S77, N80, L81, I95, R97, D114, S116, Y123, T143, K146, and W147. The only exception was the residue I124 which has previously not been implicated in peptide binding. It seems obvious that the occupation of the F pocket through EPX results in the binding of peptides with a different AA at p*Ω*. To structurally understand the impact of the F pocket occupation through EPX, we performed peptide docking using B∗15:02 bound to EXP as basis. Representative peptides sequenced from untreated (SQATPHSSY) and epoxide treated samples (VSQqKLQAEAQ) were then docked using Rosetta FlexPepDock and are illustrated in Figure 5.

## 4. Discussion

Several ADRs are associated with distinct allelic subtypes of the HLA system. The most prominent association is the abacavir-induced hypersensitivity in HLA-B∗57:01-positive patients. It could be demonstrated that the drug binding to the F pocket of B∗57:01 modifies the surface of the PBR resulting in binding and presentation of an altered peptide repertoire [5] that is the cause for severe immunogenic hypersensitivity reactions. For CBZ-mediated ADRs, the mechanism still remains unknown, although it is known that hypersensitivity reactions can occur in HLA-A∗31:01- or HLA-B∗15:02-positive patients. Clinical symptoms of B∗15:02-positive individuals suffering from CBZ-mediated ADR include SJS/TEN; the involvement of CD8+ T cells has been demonstrated [71, 72].

Peptides presented by B∗15:02 showed a characteristic length with 9-mers being the most frequent. Differences in peptide motifs for low and high binding peptides were observed, including even an altered AA frequency at pΩ. This implies that tolerance against a wide variety of peptides presented by B∗15:02 is developed during maturation of T cells. In CBZ-mediated ADRs, the drug itself or a reactive metabolite has to somehow interfere with this highly accurate recognition of self-peptides in the context of self-HLA. For the hapten/prohapten model as well as for the altered repertoire model, covalent binding of the drug to a protein that is processed and presented afterwards or binding to the HLA molecule itself is crucial, whereas the p-i model requires a transient interaction of the drug with the immune receptors. Since not only CBZ itself might cause the ADRs but also metabolic products, we focused on CBZ and its main metabolite EPX and analyzed binding of the chemicals to sB∗15:02. Affinity purification of functional trimeric HLA complexes produced in the presence and absence of the drugs and subsequent mass spectrometric quantification of CBZ and EPX revealed the ability to bind either drug. Further analysis showed that, after separation of low and high binding peptides, the peptide fractions were free of the drugs indicating binding to the HLA heavy chain.

These results emerged the question whether CBZ or its main metabolite EPX might have an effect on peptide recruitment or whether the chemicals directly interact with the HLA molecules and T cell receptors. In view of the variety of low and high binding peptides presented by both alleles, binding of CBZ might not simply alter the shape of the PBR leading to a shift in the peptide repertoire but could also induce a conformational change of the HLA molecule that is recognized by the T cells. Therefore, alterations in the B∗15:02-restricted peptide anchors following CBZ or EPX treatment of the human lymphoblastoid cell line LCL721.221 expressing sB∗15:02 molecules were evaluated.

For B∗15:02 mainly found in South Asia where it is associated with severe cases of SJS and TEN occurring averagely after 15 days [57], treatment with EPX, but not CBZ, altered peptide presentation leading to a different peptide-binding motif. Treatment with CBZ did not affect the anchor residues at pΩ, but following EPX treatment, the frequency of polar AAs glutamine and lysine was increased and replaced the nonpolar tyrosine and methionine at pΩ. Similarly, polar threonine was exchanged for nonpolar alanine at the N-terminus of the peptide. At p2, alterations after treatment with the drugs were comparable to frequencies of AAs in untreated cells described in the literature [5, 60], showing that leucine was underrepresented in untreated cells. At p3, no alterations were found. These results suggest that the trigger in HLA-B∗15:02-associated CBZ hypersensitivity is EPX. This was the first time that the effect of CBZ and EPX on peptide presentation of HLA-B∗15:02 was compared. Surface plasmon resonance measurement by Wei et al. [61] has already revealed the ability of HLA-B∗15:02 to bind not only CBZ but also EPX and other chemicals possessing a 5-carboxamide on the tricyclic ring. We showed that EPX is not only able to bind to HLA-B∗15:02 but also affect peptide presentation, leading to the assumption of the altered repertoire model for HLA-B∗15:02-associated CBZ ADRs.

Already in 2007 Yang et al. [60] have found no alterations in peptide presentation after treatment of C1R cells expressing soluble HLA-B∗15:02 with CBZ. The peptide motif described for HLA-B∗15:02 is similar to our findings. They identified C-terminal aromatic AAs tyrosine or phenylalanine and defined leucine, valine, or glutamine as peptide anchors at p2, of which leucine was underrepresented in this analysis. Additionally, Yang et al. reported an atypical preference of polar and neutral amino acids serine and threonine at nonanchoring residues p5–p8. This is in accordance with our finding when comparing AA frequencies at these positions (Sup. Tables 1 and 2).

Illing et al. [5] analyzed the impact of CBZ on peptide presentation by C1R cells expressing membrane-bound HLA-B∗15:02 and have found a much weaker change in amino acid frequencies than they did for B∗57:01 and abacavir. After treatment with CBZ, they reported unchanged anchor AAs but a higher frequency of smaller residues at p4 and p6 matching their model of CBZ binding to the D pocket of B∗15:02.

The AA frequencies are not stable after treatment with both chemicals and minor changes can be observed for low and high binding peptides in both conditions. This might be caused by the drugs binding to the HLA molecule via another additional mechanism.

B∗15:02-restricted peptides presented under the different conditions had a comparable number of modifications (40%). Nevertheless, at p4, the deamidation rate was increased after EPX treatment. It has already been described that HLA class I molecules are presenting posttranslationally modified peptides and that alterations in such modifications might form neoepitopes recognized by T cell receptors [73–77]. In celiac disease, deamidated gluten peptides have been described to be presented by certain HLA-DQ alleles [78], but until now, deamidation as a factor in HLA class I-associated diseases has not been reported. Binding of CBZ or EPX to the PBR of B∗15:02 might increase the affinity of p4-deamidated peptides. Binding of the drugs at this position would fit the model of Illing et al. [5] suggesting CBZ to bind at the D pocket of B∗15:02.

Our findings of the main metabolite EPX influencing peptide presentation of HLA-B∗15:02 are a first hint to explain the molecular mechanism of B∗15:02-associated CBZ hypersensitivity.

Illing et al. [5] have modelled CBZ binding in the PBR, assuming an interaction of CBZ with the AAs at positions 9, 62, 66, 97, 99, 156, and 159. Wei et al. [61] suggested both CBZ and/or EPX interact with arginine at position 62 of B∗15:02 but bind to the B pocket. In contrast, we found that docking of EPX using RosettaLigand suggests that it binds into the F pocket of B∗15:02, interacting predominantly with tyrosine at position 74 and serine at position 77. This binding facilitates a change in the pΩ position of peptides bound after treatment with EPX and results in a much lower frequency of tyrosine residues at this position. Docking of peptide sequences to unliganded or EPX bound homology models was used to depict the steric hindrance of bound EPX on the selection of peptides by HLA-B∗15:02 (Figure 5).

Analysis revealing CBZ-mediated ADRs being independent of polymorphisms in CYP3A4 [50] has been performed in Han Chinese population; however, a Canadian cohort that was studied showed independence of CBZ-mediated ADRs from epoxide hydrolase [59]. Epoxide hydrolase 1 (EPHX1) is an enzyme catalyzing the detoxification of EPX to carbamazepine-10,11-*trans*-diol that is urinary excreted [79, 80]. In Caucasians, the allele A∗31:01 is associated with CBZ-mediated ADRs, since B∗15:02 is not prevalent in this population [52]; thus, these results do not play a role in B∗15:02-associated CBZ hypersensitivity in Han Chinese population. Another study analyzed the association of polymorphisms in the multidrug resistance protein 1 (ABCB1), CYP3A4, EPHX1, FAS, and SCN1a in Han Chinese, where the allele B∗15:02 is common [63]. An increased risk of CBZ-mediated SJS/TEN in patients carrying the EPHX1 c.337T>C polymorphism was found. This polymorphism leads to an AA exchange at position 113 (Y113H) and has been linked to reduced expression levels and reduced or unchanged catalytic activities with different substrates in vitro [81, 82]. Contrarily, an increased carbamazepine-10,11-*trans*-diol/EPX ratio was observed in vivo in patients with a haplotype bearing the mutation [83]. Nevertheless, the influence of other mutations in the haplotype should be considered.

Therefore, we hypothesize that not metabolization of CBZ to EPX but mainly further detoxification of EPX plays a role in B∗15:02-associated CBZ hypersensitivity. A higher level of EPX might have a bigger influence than the initial dose of CBZ, since the occurrence of an ADR was independent of the CBZ dosage [63]. Modified metabolization of EPX might facilitate alterations in peptide presentation according to the altered repertoire model with EPX binding to the PBR in the F pocket.

## 5. Conclusions

The occurrence of type B ADRs often leads to severe clinical complications due to the involvement of the immune system. In the case of CBZ, this leads to the exclusion of patients from a very potent and widely used drug. Our results stress the fact that B∗15:02-associated CBZ hypersensitivity is triggered by the main metabolite EPX. This implies that not only the HLA allele and T cell receptor but also drug metabolism plays a role in the disease, and further studies on drug metabolism and/or the reaction to this metabolite may lead to additional ways to make this medication or a safely metabolized derivative accessible for these patients.

## Figures and Tables

**Table 1:** Multiple reaction monitoring (MRM) transitions monitored (*m*/*z*) with cone and collision energy.

Analyte	MRM (*m*/*z*)	Dwell (s)	Cone (V)	Collision energy (eV)
Carbamazepine	**237.1 ⟶ 194.1**	0.03	39	13
	237.1 ⟶ 179.0	0.03	39	35
Carbamazepine-D8	**245.1 ⟶ 202.1**	0.03	32	22
	245.1 ⟶ 185.0	0.03	32	36
Carbamazepine-10,11-epoxide	**253.0 ⟶ 236.0**	0.03	20	11
	253.0 ⟶ 231.2	0.03	20	27
Carbamazepine-10,11-epoxide-D8	**261.0 ⟶ 244.1**	0.03	20	12
	261.0 ⟶ 188.1	0.03	20	22

## Data Availability

The peptide data, mass spectrometric analysis, and molecular model data used to support the findings of this study are included within the article. The tables with the amino acid frequency data used to support the findings of this study are included within the supplementary information file.

## Abbreviations

AA: Amino acid
ADR: Adverse drug reaction
CBZ: Carbamazepine
DRESS: Drug reaction with eosinophilia and systemic symptoms
EPX: Carbamazepine-10,11-epoxide
EPHX1: Epoxide hydrolase 1
HLA: Human leukocyte antigen
MPE: Maculopapular eruption
PBR: Peptide-binding region
pHLA: Peptide-HLA
sHLA: Soluble HLA
SJS: Stevens-Johnson syndrome
TEN: Toxic epidermal necrolysis.

## Supplementary Materials

Supplementary Materials Sup. Table 1: amino acid frequencies in HLA-B∗15:02-restricted low binding peptides. Sup. Table 2: amino acid frequencies in HLA-B∗15:02-restricted high binding peptides. Supplementary Figure 1: fragment spectrum of deamidated peptide ARDqLVESLAQ of HLA-B∗15:02. Supplementary Figure 2: fragment spectrum of deamidated peptide VSQqKLQAEAQ of HLA-B∗15:02.
