# Supplementary material for: Carbamazepine-Mediated Adverse Drug Reactions: CBZ-10,11-epoxide but Not Carbamazepine Induces the Alteration of Peptides Presented by HLA-B∗15:02
Source: J Immunol Res. 2018 Sep 13;2018:5086503. doi: 10.1155/2018/5086503 (PMC6158965; doi:10.1155/2018/5086503)
Supplement: Supplementary Materials — Sup. Table 1: amino acid frequencies in HLA-B∗15:02-restricted low binding peptides. Sup. Table 2: amino acid frequencies in HLA-B∗15:02-restricted high binding peptides. Supplementary Figure 1: fragment spectrum of deamidated peptide ARDqLVESLAQ of HLA-B∗15:02. Supplementary Figure 2: fragment spectrum of deamidated peptide VSQqKLQAEAQ of HLA-B∗15:02. [file 5086503.f1.docx]

**
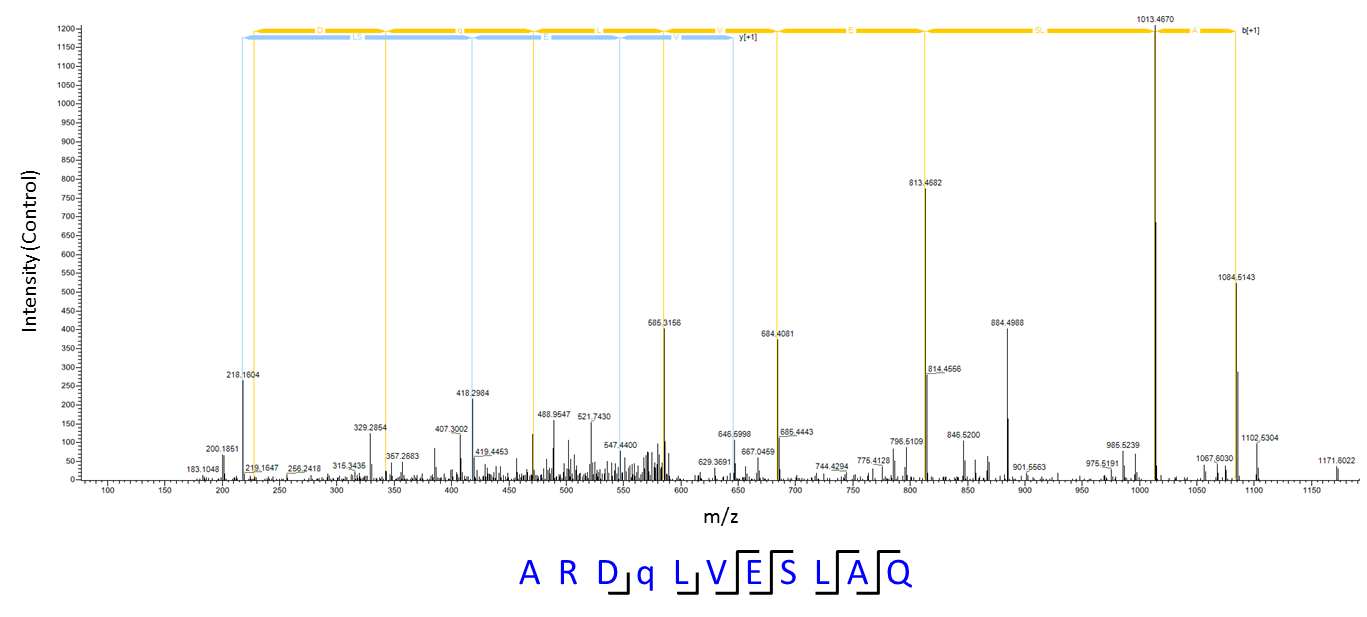
Supplementary Fig. 1: Fragment spectrum of deamidated peptide ARDqLVESLAQ of HLA-B*15:02.**

The peptide was verified by MS/MS. Coverage of y- ions (blue) and b- ions (yellow) allowed the verification of deamidation at peptide position 4.


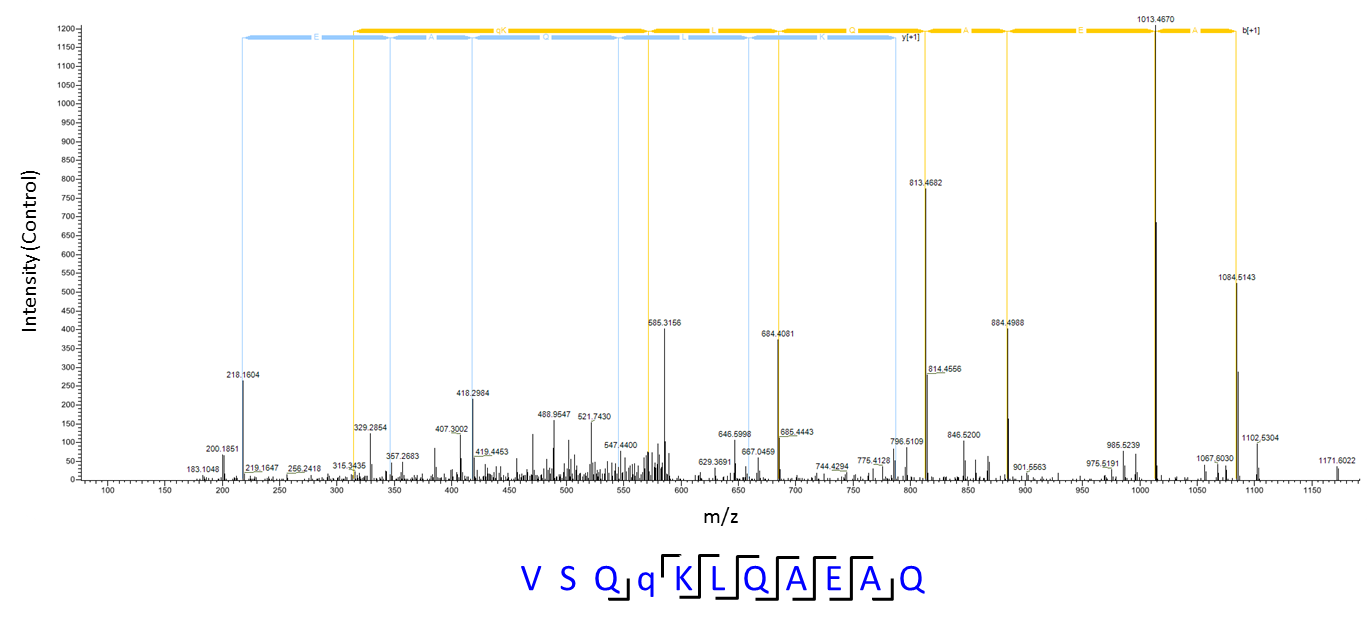


**Supplementary Fig. 2: Fragment spectrum of deamidated peptide VSQqKLQAEAQ of HLA-B*15:02.**

The peptide was verified by MS/MS. Coverage of y- ions (blue) and b- ions (yellow) allowed the verification of deamidation at peptide position 4.

**Sup. Table 1: Amino acid frequencies in HLA-B*15:02-restricted low binding peptides.**

| **AA** | **P1** | **P2** | **P3** | **P4** | **P5** | **P6** | **P7** | **P8** | **P9** | **P10** | **P11** | **PΩ** |
| --- | --- | --- | --- | --- | --- | --- | --- | --- | --- | --- | --- | --- |
| **H** | 15% | 0% | 4% | 0% | 9% | 9% | 0% | 2% | 0% | 0% | 0% | 0% |
| **K** | 4% | 0% | 13% | 4% | 9% | 2% | 4% | 9% | 0% | 0% | 0% | 0% |
| **R** | 2% | 0% | 20% | 7% | 2% | 13% | 7% | 4% | 0% | 0% | 33% | 7% |
| **D** | 9% | 2% | 0% | 9% | 0% | 13% | 0% | 0% | 0% | 0% | 0% | 0% |
| **E** | 7% | 0% | 2% | 7% | 4% | 0% | 7% | 4% | 3% | 0% | 0% | 0% |
| **C** | 0% | 0% | 0% | 0% | 0% | 0% | 0% | 0% | 0% | 0% | 0% | 0% |
| **N** | 4% | 4% | 0% | 7% | 4% | 7% | 4% | 2% | 0% | 0% | 0% | 0% |
| **P** | 0% | 4% | 7% | 15% | 2% | 0% | 9% | 4% | 0% | 0% | 0% | 4% |
| **Q** | 0% | 28% | 7% | 11% | 2% | 7% | 9% | 11% | 0% | 10% | 33% | 4% |
| **S** | 15% | 4% | 0% | 4% | 22% | 7% | 15% | 15% | 0% | 0% | 0% | 0% |
| **T** | 22% | 7% | 7% | 2% | 11% | 11% | 9% | 7% | 3% | 0% | 0% | 0% |
| **A** | 4% | 7% | 17% | 2% | 9% | 7% | 13% | 9% | 8% | 20% | 0% | 0% |
| **G** | 4% | 7% | 2% | 7% | 9% | 15% | 4% | 4% | 8% | 20% | 0% | 2% |
| **I** | 0% | 9% | 7% | 2% | 2% | 4% | 4% | 2% | 3% | 20% | 0% | 7% |
| **L** | 2% | 9% | 4% | 15% | 7% | 4% | 4% | 11% | 5% | 10% | 0% | 7% |
| **M** | 0% | 2% | 2% | 2% | 2% | 0% | 0% | 4% | 16% | 10% | 0% | 20% |
| **V** | 9% | 17% | 2% | 7% | 7% | 2% | 11% | 7% | 3% | 0% | 0% | 0% |
| **F** | 2% | 0% | 2% | 0% | 0% | 0% | 0% | 0% | 24% | 0% | 0% | 17% |
| **W** | 0% | 0% | 0% | 0% | 0% | 0% | 0% | 0% | 0% | 0% | 0% | 0% |
| **Y** | 0% | 0% | 4% | 0% | 0% | 0% | 0% | 4% | 29% | 10% | 33% | 33% |

**Sup. Table 2: Amino acid frequencies in HLA-B*15:02-restricted high binding peptides.**

| **AA** | **P1** | **P2** | **P3** | **P4** | **P5** | **P6** | **P7** | **P8** | **P9** | **P10** | **P11** | **PΩ** |
| --- | --- | --- | --- | --- | --- | --- | --- | --- | --- | --- | --- | --- |
| **H** | 8% | 0% | 0% | 0% | 3% | 11% | 0% | 0% | 0% | 0% | 0% | 0% |
| **K** | 3% | 0% | 5% | 0% | 11% | 5% | 5% | 8% | 0% | 7% | 0% | 11% |
| **R** | 3% | 0% | 11% | 5% | 0% | 0% | 11% | 14% | 0% | 7% | 11% | 8% |
| **D** | 0% | 5% | 0% | 5% | 5% | 5% | 0% | 0% | 0% | 0% | 0% | 0% |
| **E** | 14% | 5% | 0% | 8% | 3% | 8% | 3% | 5% | 6% | 13% | 0% | 3% |
| **C** | 0% | 3% | 0% | 0% | 0% | 0% | 0% | 0% | 0% | 0% | 0% | 0% |
| **N** | 5% | 0% | 5% | 5% | 3% | 5% | 5% | 0% | 3% | 0% | 0% | 0% |
| **P** | 0% | 3% | 8% | 11% | 14% | 0% | 5% | 5% | 0% | 7% | 0% | 3% |
| **Q** | 0% | 14% | 3% | 14% | 5% | 11% | 8% | 5% | 0% | 0% | 22% | 5% |
| **S** | 22% | 5% | 3% | 3% | 11% | 11% | 16% | 19% | 0% | 0% | 0% | 0% |
| **T** | 19% | 11% | 3% | 5% | 11% | 14% | 11% | 8% | 3% | 7% | 11% | 0% |
| **A** | 3% | 11% | 24% | 11% | 8% | 11% | 5% | 11% | 9% | 13% | 22% | 3% |
| **G** | 5% | 11% | 3% | 8% | 11% | 8% | 5% | 3% | 0% | 20% | 0% | 8% |
| **I** | 0% | 3% | 5% | 5% | 0% | 3% | 8% | 3% | 3% | 0% | 0% | 0% |
| **L** | 5% | 8% | 3% | 8% | 14% | 5% | 5% | 11% | 12% | 0% | 11% | 0% |
| **M** | 0% | 3% | 0% | 0% | 0% | 0% | 0% | 0% | 15% | 0% | 0% | 11% |
| **V** | 11% | 19% | 16% | 8% | 3% | 3% | 5% | 8% | 3% | 0% | 22% | 3% |
| **F** | 3% | 0% | 5% | 3% | 0% | 0% | 0% | 0% | 21% | 0% | 0% | 16% |
| **W** | 0% | 0% | 0% | 0% | 0% | 0% | 3% | 0% | 0% | 0% | 0% | 0% |
| **Y** | 0% | 0% | 5% | 0% | 0% | 0% | 3% | 0% | 26% | 27% | 0% | 30% |

Supplementary Figure 1: Fragment spectrum of deamidated peptide ARDqLVESLAQ of HLA-B*15:01.

The peptide was verfied by MS/MS. Coverage of y- ions (blue) and b- ions (yellow) allowed the verification of deamidation at peptide position 4.
